# Isolated Central Nervous System Relapse in Chronic Myeloid Leukemia

**DOI:** 10.1155/2015/232915

**Published:** 2015-03-23

**Authors:** Juliana Gomez, Victor Duenas

**Affiliations:** ^1^Internal Medicine Department, Guillermo Almenara Irigoyen National Hospital, Grau Avenue 800, Lima, Peru; ^2^National Institute of Neurologic Sciences, Ancash Street 1271, Barrios Altos, Lima, Peru

## Abstract

Chronic myeloid leukemia is a myeloproliferative disorder that has three distinguished phases: chronic, accelerated, and blastic. In extremely rare cases, the blast phase can affect the central nervous system without concomitant bone marrow involvement. We report the case of a patient with chronic myeloid leukemia who, despite having achieved complete cytogenetic remission in the bone marrow for several years, experienced a blast crisis of the central nervous system following an episode of infectious meningoencephalitis.

## 1. Introduction

Chronic myeloid leukemia (CML) is a myeloproliferative disorder characterized by the presence of a reciprocal translocation between chromosomes 9 and 22, known as the Philadelphia chromosome, which results in expression of an oncoprotein, termed BCR-ABL [1, 2]. CML has 3 phases: chronic, accelerated, and blast phase [3]. Blast phase (BP) or blast crisis is defined as the presence of 20% or more blasts in peripheral blood or bone marrow (BM), or a large focus of blasts in BM, or an extramedullary blast proliferation [4]. The central nervous system as a site of extramedullary blast crisis is extremely rare [5], and when affected it usually occurs concurrently with systemic relapse [6].

We here describe the case of a patient with chronic myeloid leukemia who, after an episode of infectious meningoencephalitis, experienced blast crisis of the central nervous system although having achieved complete cytogenetic remission in the bone marrow for several years with imatinib treatment.

## 2. Case Presentation

The patient was a 33-year-old Hispanic man who had a history of Philadelphia-positive CML, diagnosed in the chronic phase 5 years ago. After diagnosis, he received treatment with imatinib 400 mg QD, demonstrating complete hematological and cytogenetic bone marrow response within 4 months of therapy initiation. This treatment was maintained in the following years without relapse. The patient presented to the emergency department having experienced an eight-day history of severe headaches, nausea, and confusion and 3 days of low-grade fever. Upon direct examination the patient was febrile (100.4°F), tachycardic, and lethargic, showed nuchal rigidity, and had 3 episodes of tonic-clonic seizures. The patient did not present with evidence of lymphadenopathy or hepatosplenomegaly. Head and thoracoabdominopelvic computed tomography scans were normal. WBC count was 12 960/*μ*L (75% neutrophils, 3% bands, 4% monocytes, and 18% lymphocytes). CSF showed 1010 cells/*μ*L (100% mononuclears), glucose of 41 mg/dL (N: 40–76), and proteins of 29 mg/dL (N: 15–45); Gram stain and HIV antibody detection were negative. PCR testing of the CSF and viral culture for herpes simplex were not available at our institution. The patient received treatment with ceftriaxone, vancomycin, acyclovir, mannitol, and anticonvulsants. Imatinib was discontinued thereafter. Five days later the patient's symptoms improved, he was alert, oriented, and afebrile, and the leukocytosis resolved. He subsequently was managed with acyclovir and anticonvulsants after obtaining negative bacterial CSF and blood cultures.

**Figure 1:**
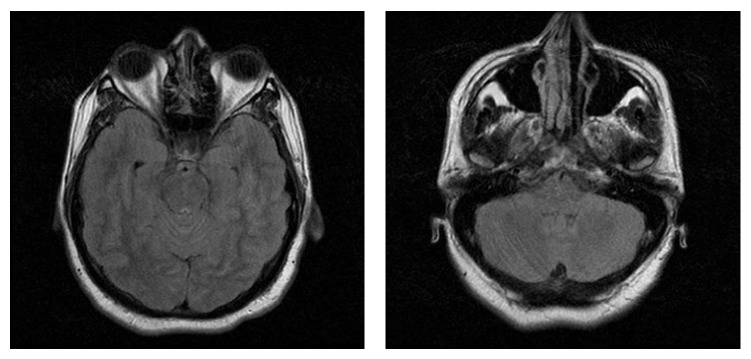
Brain MRI in FLAIR sequence, without abnormalities.

Eighteen days after admission the patient again developed severe headaches, vomit, confusion, and worse nuchal rigidity. A brain magnetic resonance imaging (MRI) scan was normal (Figure 1). BM aspiration assessment indicated complete hematologic, cytogenetic, and molecular remission. New CSF study showed 760 cells/*μ*L (100% mononuclears), glucose of 24 mg/dL, and proteins of 79 mg/dL, and cytocentrifuge preparations lead to cytological identification of leukemic blasts.

**Figure 2:**
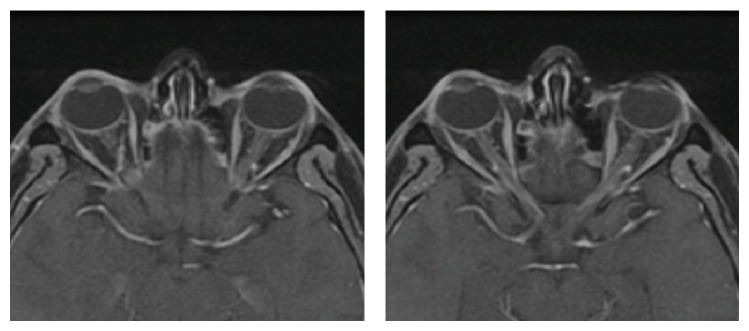
Optic MRI without abnormalities.

CSF cytogenetic study showed 46XY, t(9;22)(q34:q11). Fluorescence in situ hybridization (FISH) analysis detected BCR-ABL fusion signals in 4.1% International Scale (IS) of cells. Flow cytometry to determine the blast type and measurement of imatinib concentration in serum and CSF were not available at our institution at the time. One month following admission, the patient received intrathecal methotrexate (12 mg), cytosine arabinoside (30 mg), and dexamethasone (4 mg), a total of 4 doses in a month. Few days after the first dose, the neurological symptoms improved substantially and the patient was able to initiate dasatinib 70 mg BID PO. However, when fully oriented, the patient manifested decreased visual acuity. Funduscopy showed pale discs without edema suggestive of optic nerve atrophy. Optic MRI did not evidence abnormalities (Figure 2). After a few weeks the patient achieved full recovery except for the persistence of severe visual deficit. CSF became negative for blasts after 2 weeks of intrathecal therapy. The patient was discharged on dasatinib treatment. Follow-up at 6 months after discharge indicated the patient remained in complete cytogenetic response without signs of systemic or central nervous system (CNS) relapses; unfortunately, the severe visual impairment persisted.

## 3. Discussion

Chronic myeloid leukemia is a myeloproliferative neoplasm with an incidence of one-two cases per 100 000 adults and accounts for 15% of newly diagnosed cases of leukemia in adults in the USA [7]. About 30 to 50% of CML patients are asymptomatic at diagnosis, in whom the disease is found on routine physical examination or blood tests. CML can be classified into chronic phase, accelerated phase, and blast phase. Diagnosis is most commonly made during the chronic stage and, if present, the symptomatology of this phase is mainly the result from anemia and splenomegaly. Lymphadenopathy, constitutional symptoms, and infiltration of skin or other tissues are uncommon. When present, they favor Philadelphia-negative CML or accelerated phase or blast phase of CML. Accelerated phase might be insidious or present with worsening anemia, splenomegaly, and organ infiltration. Most patients evolve into accelerated phase before BP, but 20% transit directly into BP, which usually presents with worsening constitutional symptoms, bleeding, fever, and infections [3]. In 5–10% of the cases, the blast phase can present at extramedullary sites [2]. Lymph nodes, serosal surfaces, skin and soft tissue, breast, bone, and gastrointestinal or genitourinary tract are among the principal areas involved [8]. The central nervous system as a site of extramedullary blast crisis is quite rare [5].

In previously reported cases of CML with isolated CNS blast crisis, the main presenting symptom was headache with or without other neurologic symptoms. The majority of patients had complete cytogenetic response state at CNS relapse and the median time from treatment initiation to CNS relapse was 24 months with a range of 3 to 58 months [5, 8–22].

Imatinib, a BCR-ABL tyrosine kinase inhibitor, is capable of inducing major or complete cytogenetic responses in most CML patients [23, 24], changing dramatically the 10-year overall survival from 20 to 80–90% [7, 25]; however, it has been documented that it penetrates poorly into the CSF [6, 11, 26], probably due to increased efflux of the drug from the CNS due to P-glycoprotein [27]. This emphasizes the problem of imatinib pharmacokinetics in the CNS, which might be an important issue to consider in patients taking the drug for over a year [12].

It has been suggested that CML patients with complete cytogenetic response following imatinib regimen and with history of any other CNS disease may need prophylaxis against CML expansion in CNS. Isobe et al. reported a case similar to ours of a male patient with CML in complete cytogenetic response for less than a year who developed CNS relapse after discontinuation of imatinib intake for over 6 weeks due to complications of viral meningitis [11]; however, our patient was in remission for over 4 years and the discontinuation of the drug was around 3 weeks when he developed symptoms of CNS relapse. Identifying which conditions may increase the risk of CNS involvement after receiving imatinib therapy in CML patients could be very helpful in the future [10, 11].

In the last decade there have been several reports of CML patients with CNS BP while on imatinib therapy; however, no surveillance strategy for CNS relapse or treatment guidelines for documented CNS relapse in this kind of patients have yet been defined.

Although most cases of CNS BP have been treated with triple intrathecal chemotherapy [5, 8, 9, 12, 15], dasatinib, a second generation multityrosine kinase inhibitor with much greater potency (325-fold) over imatinib [28, 29], has also been used, in addition to chemotherapy, for that purpose in some cases reported, as well as for maintenance treatment, with good outcomes [9, 10, 14, 15, 28].

Unfortunately, our patient was left with significant visual sequela, probably due to chronic intracranial hypertension and/or optic nerve leukemic infiltration; if CNS BP had been suspected and diagnosed earlier, perhaps this complication could have been prevented or properly treated. This highlights the fact that physicians must have a high suspicion index for CNS infiltration in patients with a CML history who manifest headache and meningismus even after achieving complete cytogenetic and molecular BM response with imatinib.
